# Expression Regulation of Polycistronic *lee3* Genes of Enterohaemorrhagic *Escherichia coli*

**DOI:** 10.1371/journal.pone.0155578

**Published:** 2016-05-16

**Authors:** Wei-Sheng W. Sun, Jenn-Wei Chen, Yi-Chih Wu, Hsing-Yuan Tsai, Yu-Liang Kuo, Wan-Jr Syu

**Affiliations:** 1 Institute of Biochemistry and Molecular Biology, National Yang-Ming University, Taipei, Taiwan, R.O.C; 2 Taiwan International Graduate Program in Molecular Medicine, Academia Sinica, Taipei, Taiwan, R.O.C; 3 Department of Microbiology and Immunology, National Cheng-Kung University, Tainan, Taiwan, R.O.C; 4 Institute of Microbiology and Immunology, National Yang-Ming University, Taipei, Taiwan, R.O.C; 5 Department of Medical Imaging and Radiological Sciences, Chung Shan Medical University, Taichung, Taiwan, R.O.C; 6 Department of Medical Imaging, Chung Shan Medical University Hospital, Taichung, Taiwan, R.O.C; National University of Singapore, SINGAPORE

## Abstract

Enterohaemorrhagic *Escherichia coli* O157:H7 (EHEC) carries a pathogenic island LEE that is consisted mainly of five polycistronic operons. In the *lee3* operon, *mpc* is the first gene and has been reported to down regulate the type-3 secretion system of EHEC when its gene product is over-expressed. Furthermore, *mpc* has been suggested to have a regulation function via translation but the mechanism remains unclear. To clarify this hypothesis, we dissected the polycistron and examined the translated products. We conclude that translation of *mpc* detrimentally governs the translation of the second gene, *escV*, which in turn affects the translation of the third gene, *escN*. Then sequentially, *escN* affects the expression of the downstream genes. Furthermore, we located a critical *cis* element within the *mpc* open-reading frame that plays a negative role in the translation-dependent regulation of *lee3*. Using qRT-PCR, we found that the amount of *mpc* RNA transcript present in EHEC was relatively limited when compared to any other genes within *lee3*. Taken together, when the transcription of LEE is activated, expression of *mpc* is tightly controlled by a restriction of the RNA transcript of *mpc*, translation of which is then critical for the efficient production of the operon’s downstream gene products.

## Introduction

*Enterohaemorrhagic Escherichia coli* (EHEC) is a food-borne bacterium that causes abdominal cramps, diarrhea and haemorrhagic colitis. To children and the elderly, the infection may further develop into hemolytic uremic syndrome (HUS) and lead to serious renal failure and hemolytic anemia [1]. Ingestion of contaminated food is the most common reason for EHEC infection. The cattle intestine is regarded as the main reservoir of EHEC, a reasoning that pinpoints raw or under-cooked ground beef and raw milk as frequent sources of EHEC infection outbreaks. However, this bacterium is increasingly being identified as also associated with contaminated fruits and vegetables [1–3].

To cause diseases, EHEC possesses virulence factors to result in pathogenesis. Shiga-like toxins, the major cause of the aforementioned HUS, are encoded by a prophage embedded in the EHEC chromosome [4]. There are other EHEC virulence factors that are encoded by a gene cluster located on the chromosome. In this regard, they include a type-three secretion system (T3SS), translocators, and effector proteins [1]. T3SS contains a needle-like device that helps the delivery of bacterial effector proteins into host cells. The structure of the secretion device can be divided roughly into the basal apparatus, the needle and the translocation pore [5]. The basal apparatus is a structure spanning across bacterial inner membrane and outer membrane. It is composed of an outer membrane ring, an inner membrane ring and an inner rod, through which the secretion of needle structure subunits, translocators and effector proteins is made possible [5]. The needle structure is a hollow, stiff tube consisting of EscF, which polymerizes to span between inner and outer membranes, and polymerized EspA that forms a sheath-like structure and extends outward from EspF; and this structure allows the passage of unfolded secretory proteins [5, 6]. EspA, EspB and EspD are secreted through T3SS and function as the translocators. When they contact a host cell, EspB and EspD form complexes in the needle tip of the EspA structure that allow the formation of pores through the plasma membrane of the contacted eukaryote host cell [5]. The effector proteins, such as Tir and Map, are then secreted through this system into host cells in order to modulate various physiological activities including cytoskeleton rearrangement, disruption of tight junctions and pedestal-like structure formation [7].

The EHEC proteins associated with T3SS are mainly encoded by a cluster of genes on a pathogenic island named the Locus of Enterocyte Effacement (LEE). LEE consists of 41 open reading frames (ORFs), most of which are distributed among five operons (*lee1* to *lee5*) [8]. In addition to the structural proteins, translocators, and effector proteins, LEE encodes other proteins that act as regulators and chaperons. Ler is the global regulator of LEE and is encoded by the 1^st^ gene of the *lee1* operon. It regulates the transcription activation of the *lee2*, *lee3*, *lee4*, *lee5* and *grlRA* operons [9]. In addition to regulating LEE, Ler has been reported to activate genes outside of LEE [10, 11]. GrlR and GrlA are products of a bi-cistronic operon located between *lee1* and *lee2*. GrlA regulates LEE by directly binding to *lee1* promoter and enhances expression of *ler* [12]. On the other hand, GrlR acts by binding to GrlA and suppresses the activation effect of GrlA on *lee1* [12]. However, GrlA and GrlR have been identified as having an opposite regulation mechanism during flagellar-gene expression [13].

Mpc, encoded by the first open reading frame (ORF) of *lee3*, is able to interact with Ler and represses the activity of Ler when over-expressed. Furthermore, when an EHEC mutant carries an initiation codon mutation (an A to C substitution at the 1^st^ nucleotide of the codon) within *mpc*, abolishment of bacterial T3SS expression has been observed. However, restoration of T3SS activity in this strain (named AC36) occurs simply by complementing this mutation with five genes downstream in the *lee3* operon, without the need of *mpc* [14]. Based on these observations, one interpretation is that Mpc is likely to be needed only in a minute amount. Equally important is the fact that Mpc expression is able to modulate the translation of downstream genes, including the gene products needed for membranous apparatus assembly [14]. An examination of the genes within *lee3* indicates that *mpc* is followed by *escV*, *escN*, *escA*, *escP*, *sepQ*, and *espH*. EscV, an inner membrane protein, is one of the structural proteins involved in building the basal apparatus [15]. EscN is highly conserved among all T3SSs and functions as ATPase, the activity of which is to hydrolyze ATP in order to release secretory proteins from chaperones. Furthermore, oligomerization of EscN is required for its optimal activity [16]. EscA has been shown to be able to interact with EspA and increase the stability of intracellular EspA [17]. In enteropathogenic *E*. *coli* (EPEC), EscP is involved in regulating the length of the T3SS needle and its assembly [18]. SepQ is not well characterized in EHEC and EPEC. However, SpaO, a homolog of SepQ in *Salmonella* Typhimurium, is known to form a cytoplasmic sorting platform that facilitates the docking of appropriate secretory proteins and chaperones [19]. The last gene product encoded by *lee3* is EspH, which plays a pivotal role in the efficient formation and elongation of the pedestal-like structure in EPEC [20].

Translational coupling is a phenomenon referring to the dependence of downstream gene translation to the translation of upstream gene [21, 22]. This phenomenon is common in many bacterial operons to coordinate the production of functionally relevant genes [23–25]. Since *lee3* are consisted of seven genes required for proper assembly of T3S apparatus, and the lost phenotype of AC36 could be recovered by exogenously expressed downstream genes, it seems reasonable to speculate that translational coupling is involved in the expression coordination [14]. However, the real status of translational coordination among the genes of *lee3* remains elusive. Recently, studies have revealed that there are interactions as well as functional coordination between some proteins encoded by *lee3* [17, 26]. Taking together, these observations suggest that the regulation of *lee3* gene expression must be sophisticated and well-organized.

The currently known aspect of the regulation of *lee3* involves functions associated with the positive factor Ler [27] and the negative factor H-NS [28]. Here, we demonstrate that post-transcriptional regulation is also important. Firstly, translation of Mpc is detrimental to the expression of downstream genes and a *cis* element within the first 100 nucleotides of *mpc* plays an important role in this regard. Secondly, the low level of Mpc in the bacterial cell would seem to be controlled by the availability of mRNA since it was found that the amount of mRNA responsible for *mpc* is significantly lower than that for any other ORFs within the same *lee3* operon.

## Material and Methods

### Bacterial strains and culture conditions

The AC36 strain of EHEC O157:H7 [14] and its parental strain (ATCC 43888, also referred as the wild-type strain here, were used in this study and the extracted chromosomal DNA of the bacteria was used as the template when amplifying the *lee3* fragments. *E*. *coli* K-12 strain JM109 (New England Biolabs) was used as the host strain during plasmid construction and protein expression. Bacteria were routinely grown at 37°C aerobically in Luria-Bertani (LB) broth (Difco). To induce T3SS activation, an overnight-culture of EHEC in LB was 1:50 diluted into M9 minimal medium (Difco) in the presence of 5% CO_2_ [29]. Ampicillin was added to the media at 100 μg ml^-1^ when necessary [29].

### Primers and expression plasmids

To evaluate the effect of translation initiation of one gene on the expression of downstream genes, appropriate expression cassettes were constructed by PCR cloning. And the ATG start codon was on purpose replaced with CTG during the 5’-end primer design in case of that the translation of the leading gene was to be abolished. All primers used in this study are listed in S1 Table. pACYC177 (New England Biolabs) was first modified by sub-cloning a fragment covering the *T5* promoter to the His_x6_-coding sequence from pQE60 (Qiagen) into the *Xho*I/*Hind*III-restricting sites; this resulted in pACYC177-*T5*-His_x6_. To construct plasmids expressing the *lee3* genes in various segments, gene fragments were amplified from the EHEC chromosomal DNA using appropriate primers. The DNA fragments were then digested with *Nco*I and *Bgl*II, which restricted the sites that were engineered at the 5’ end of the forward and the reverse primers, respectively. Each digested DNA fragment was then ligated into the pACYC177-*T5*-His_x6_ cut with the same restriction enzymes; this produced a number of constructs carrying various lengths of *lee3* fragments with the last gene being in-frame fused with the His_x6_-coding sequence. To mutate the start ATG codon to CTG of a given *lee3* gene, the desired nucleotides had been incorporated into the primer that was subsequently used in the PCR reaction. To over-express proteins, pQE60-*mpc*, pQE60-*escN*, pQE60-*escA*, and pQE60-*escP* were prepared using a strategy similar to that described above except that different appropriate primer pairs were used and the PCR products were cloned into pQE60 (Qiagen) cut with the restriction enzymes *Nco*I and *Bgl*II.

### Constructing the pM-V-N variants

Site-directed mutagenesis was carried out as described previously [30]. In brief, to generate pM^G72stop^-V-N, pM-V-N was used as template for PCR amplification with two primer pairs, RBS-F/*mpc*-G72stop-F and *mpc* G72stop-F/pACYC177-R. The so-amplified two PCR products were mixed and heated to anneal as a template for the second PCR, in which primers RBS-F and pACYC177-R were used. The second PCR gave a full-length segment covering *mpc* to *escN* except that the 214^th^ nucleotide G was replaced with T. The product was then digested with *Nco*I and *Bgl*II and ligated into the same enzyme-restricted pACYC177-*T5*-His_x6_.

Internal deletion variant of pM-V-N, i.e., p-*mpc* ^Δ51–100^ *-*V-N, was constructed by removing nucleotides 51–100 of *mpc* in p-^*AC*^M-V-N. Primers *mpc*-trunc51-100-F and *mpc*-trunc51-100-R were first phosphorylated with T4 PNK (New England Biolabs) and then used to PCR amplify the template of p-^*AC*^M-V-N. The PCR product was then self-ligated. Additional pM-V-N variants were constructed similarly with the same strategies.

### Proteins expression and Western blotting analysis

Plasmids harboring the *lee3* fragments were transformed into JM109 and the transformants were cultured at 37°C with agitation overnight. Each culture was then 1:50 diluted with fresh LB-ampicillin and cultured for a further 1.5 h. At this point IPTG (Sigma-Aldrich) was added to the culture at 0.5 mM and the culture was incubated for a further 2 h. Bacteria were then harvested and boiled directly in the SDS sample buffer (80 mM Tris-HCl, pH 6.8, 2% SDS, 0.1% bromophenol blue, 10% glycerol, 2% β-mercaptoethanol). To analyze the EHEC-secreted proteins, T3SS was activated by culturing the various EHEC strains in M9 in the presence of 5% CO_2_ for 6 h. The bacteria were then pelleted in order to analyze for the presence of proteins in the bacterial lysates, while the culture supernatants were collected and treated with trichloroacetic acid (J.T Baker) to precipitate any proteins present for similar analysis.

All proteins were analyzed by SDS PAGE, which was followed by Western blotting as previously described [30]. Proteins fused with the His_x6_ tag were detected using commercial rabbit anti-His_x6_ antibodies (Bethyl), whereas Tir, EspB, EspA, and OmpC were detected using specific antisera as described previously [14]. The immune-reacted membrane was finally developed using Western Lightning™ Chemiluminescence Reagent Plus (PerkinElmer) and the signals were captured by exposing the membranes to X-ray film (Fuji).

### RNA isolation and real-time quantitative RT-PCR (qRT-PCR)

RNA isolation was carried out as previously described [31]. In brief, total RNA was extracted using TRIzol reagent (Invitrogen) from EHEC at different time points after being switched from LB to M9. After extraction, the RNA was treated with DNase I (BioTools) to remove any contaminated chromosomal DNA and then cleaned-up using a RNA mini-prep column (Zymo Research). After determining the quality and concentration of the preparation by measuring the OD_260/280_, the RNA was reverse transcribed into cDNA using a ToolsQuant II Fast RT kit (BioTools). Real-time qRT-PCR was carried out in triplicate using the synthesized cDNA, SYBR FAST (KAPA Biosystems) and appropriate primers (S1 Table) and the ABI StepOne Plus™. Comparative Ct (ΔΔCt) system was used to determine the relative fold changes in mRNA expression levels of the various *lee3* gene; this was done by first normalizing to the level of an internal control (16S rRNA) for each individual samples and then calculating the fold change against a reference (the *mpc* RNA level obtained from the first-hour sample). To avoid a possible bias generated by the differences of primer efficiency at different regions, qPCR was also performed on an equal amount of EHEC chromosomal DNA. The efficiencies of individual primer pairs were then obtained. Thereby, the above calculated qRT-PCR results were further calibrated against the primer efficiency differences. Finally, *p* values between paired data were calculated by Student’s *t* test (*, *p* < 0.05; **, *p* < 0.01).

## Results

### Translation of *mpc* is required for the expression of the downstream genes *escV* and *escN*

**Fig 1:**
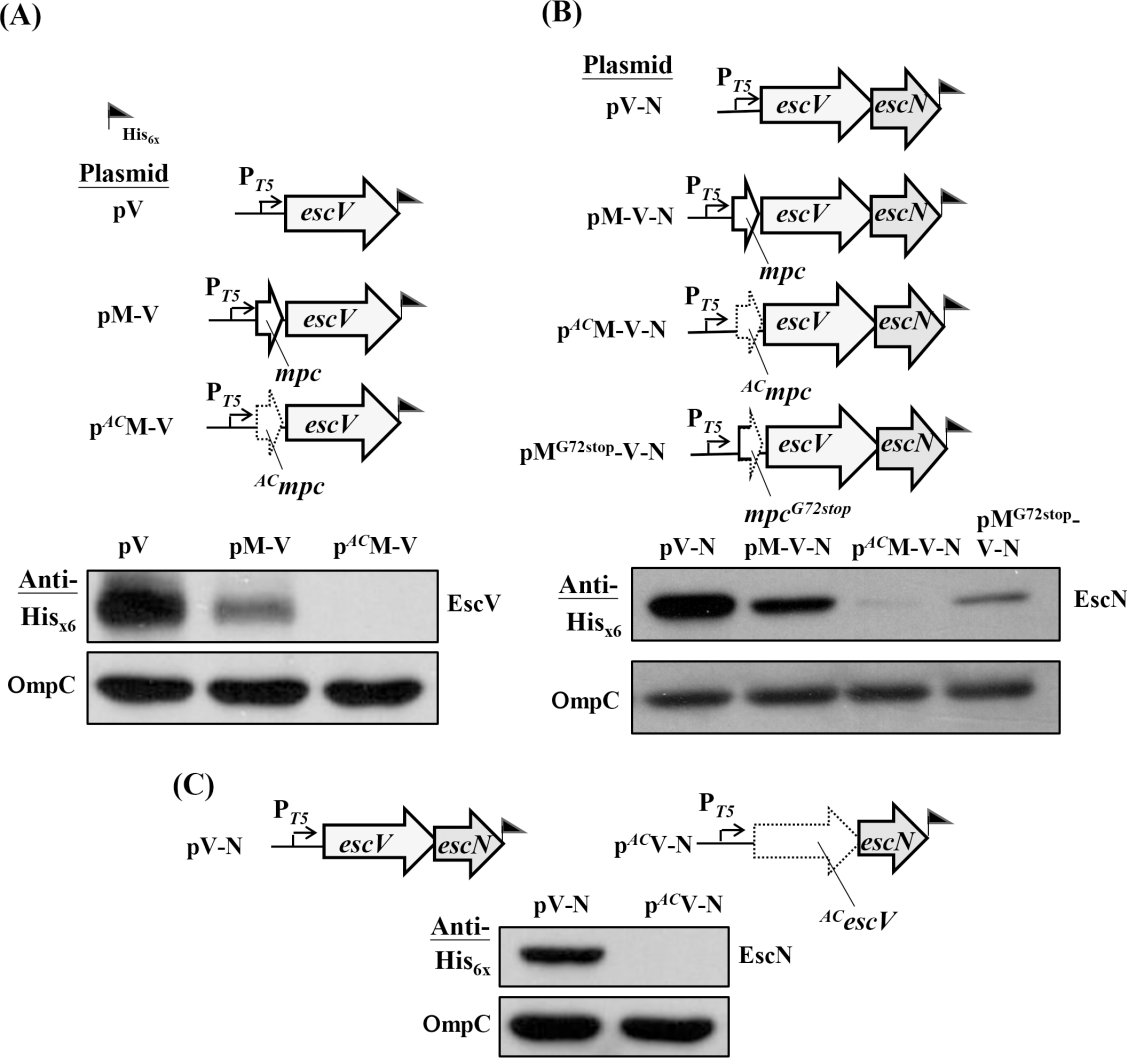
Translation of *mpc* is required for effective translation of the downstream *escV* and *escN* genes. (A) Mutating the initiation codon of *mpc* disturbs the translation of the *escV* gene product. (B) Appropriate translation of *mpc* is necessary for the detection of the *escN* gene product. (C) Translation of *escV* affecting the level of EscN produced. In all panels, illustrations of the expression plasmids are shown above the Western blotting analyses of total bacterial lysates, which were analyzed by SDS PAGE followed by immunoblotting with anti-His_x6_ tag. Plasmids were individually transformed into JM109 and the obtained transformants were cultured in LB and induced to allow protein expression by treating with IPTG for 2 h. ORFs with a mutated start codon, in which ATG was changed to CTG, are indicated by a dotted, arrowed frame whereas ORFs with an authentic configuration are framed with solid lines. A mixed-type of frame for *mpc* illustrates a premature translation with a termination codon replacing the one originally codes for Gly_72_. A flag indicates where the His_x6_ tag is introduced. For easy reading, a plasmid or ORF carrying the mutated start codon is superscripted with *AC*. Outer membrane protein OmpC was detected in each bacterial lysate using anti-OmpC antibody and this was used as a sample loading control.

To understand how the regulation mechanism of *mpc* acting on *lee3* in details, we first created three constructs (Fig 1A, upper panel) that carried *escV* (namely pV), *escV* along with upstream *mpc* (namely pM-V), and *escV* along with start-codon-altered *mpc* (namely p^*AC*^M-V) in order to compare the expression levels of EscV under these three settings. Since all transcripts were equally driven by the same *T5* promoter and EscV was tagged with His_x6_, we compared the expressed levels of EscV side-by-side by Western blotting using anti-His_x6_. The lower panel in Fig 1A shows that both pV and pM-V were able to express fair amounts of EscV, but p^*AC*^M-V appeared to yield a relative low level of EscV. Thus, *mpc* positioned before *escV* apparently attenuates the expression of *escV*. Furthermore, when the translation initiation of *mpc* was disrupted (by mutating the gene from ATG to CTG in p^*AC*^M-V), EscV was hardly detectable. This finding indicates that effective translation of *mpc* is critical for the expression of downstream *escV*.

To address whether the effective translation of *mpc* is essential to the expression of downstream gene *escN*, which is in the third position within the operon, we used a similar strategy to construct another four constructs (Fig 1B, upper panel); the first three were pV-N carrying *escV* followed by *escN*, pM-V-N covering *mpc*, *escV*, and *escN*, and p^*AC*^M-V-N that is identical to pM-V-N except for an A-to-C change of the *mpc* 1^st^ nucleotide. In these constructs, EscN was tagged with His_x6_ and could be detected by Western blotting as outlined above. Apparently, the positioning of *mpc* before *escV* had a small effect on the expression level of EscN (Fig 1B, lower panel; compare pV-N and pM-V-N). However, the level of EscN decreased greatly in the setting of p^*AC*^M-V-N. Therefore, the effective translation initiation of *mpc* also appeared to be critical to the expression of the downstream third gene.

To test whether *mpc* translation terminates prematurely could have any effect on the expression of the downstream genes, we constructed the fourth construct of pM^G72stop^-V-N (Fig 1B) by introducing a stop codon to replace the *mpc* 72^th^ codon originally coding for Gly. Fig 1B (lower panel) shows that the EscN expression level with pM^G72stop^-V-N was apparently higher than that seen with p^*AC*^M-V-N but lower than that of pM-V-N. These results suggest that an effective translation of *mpc* is readily necessary for efficient expression of downstream genes and a premature termination of *mpc* still led to the following execution inefficiently.

In the case of p^*AC*^M-V (Fig 1A), it seemed to be important that an upstream gene may affect immediate downstream gene expression. Therefore, we examined whether the effective translation of *escV* is necessary for the expression of the immediate downstream gene *escN*. This was done by creating two constructs, one of which pV-N carried a segment of the regular *escV* followed by *escN*, while the other, p^*AC*^V-N, carried an identical stretch except for an A-to-C alteration of the *escV* 1^st^ nucleotide. Western blotting analysis was used to detect the His_x6_-tagged EscN as shown in Fig 1C, which indicates that EscN can hardly be detected in p^*AC*^V-N when compared to the situation when pV-N is examined. Therefore, translation initiation of *escV* is crucial for the expression of immediately downstream *escN*. Taken together, the above findings suggest that initiation of *mpc* is necessary for an efficient translation of *escV* and that translation of this gene in turn affects the expression of downstream *escN*.

### Expression of *escA* influenced by translation of *mpc*

**Fig 2:**
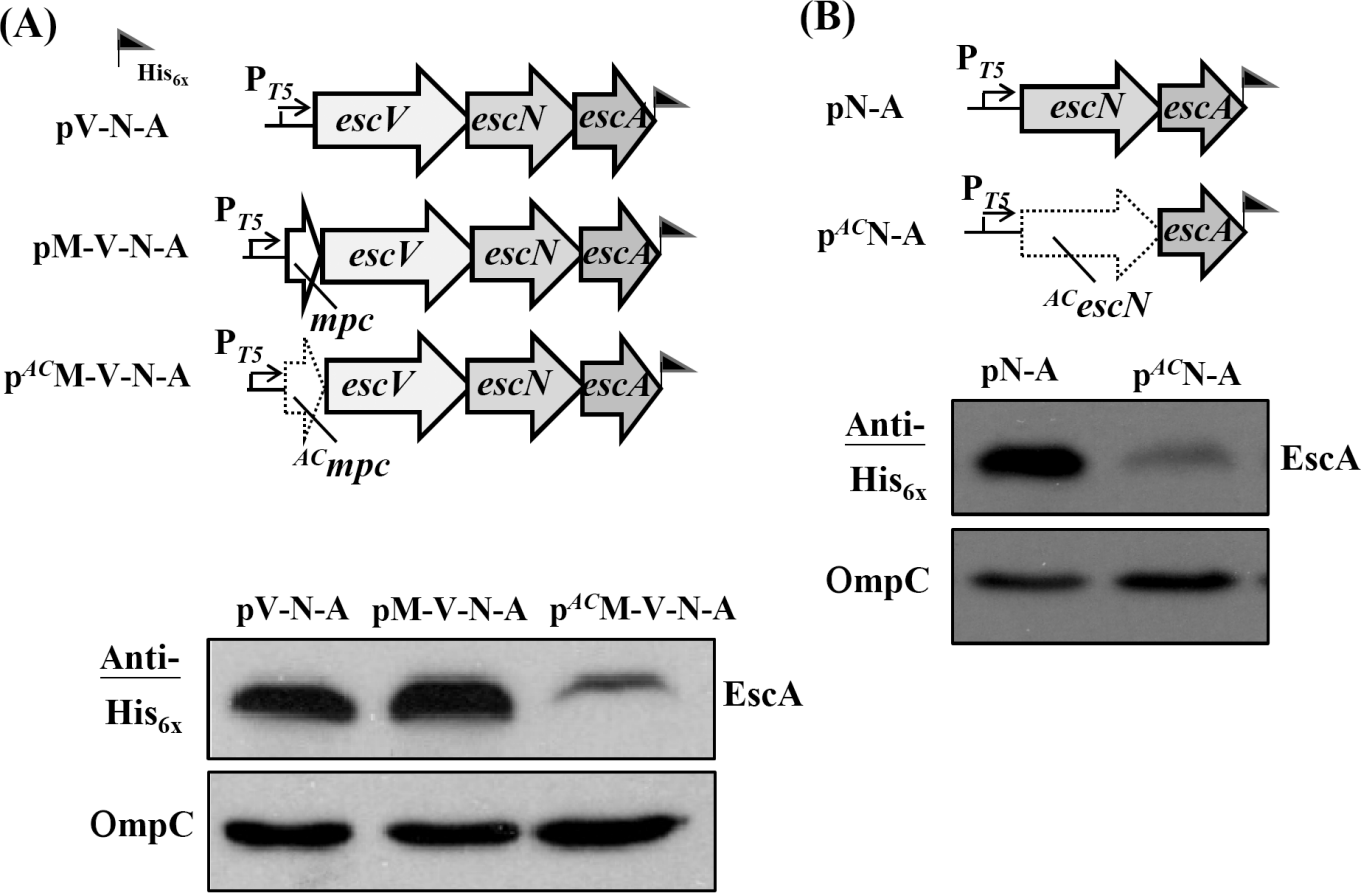
Expression of the 4^th^ gene (*escA*) of *lee3* is affected by the translation of *mpc*. Abolishing the initiation codon of *mpc* reduced the expression level of EscA. (B) Translation of *escN* affects the immediate downstream translation of *escA*. The illustrations show the plasmids together with the Western blotting analyses and these are displayed in a manner similar to that described in legend to Fig 1. OmpC from the various individual samples was detected in parallel to ensure comparable sample loadings.

To study further the influence of *mpc* translation initiation on the products of downstream genes, the expression of *escA* (the fourth ORF in *lee3*) was similarly examined. Fig 2A (upper panel) shows the three constructs that would all give a possible immuno-detected EscA with the C-terminal tag of His_x6_. pV-N-A has an expression cassette containing *escV*, *escN* and *escA*, pM-V-N-A is a construct with additional *mpc* in front of *escV*, whereas p^*AC*^M-V-N-A is identical to pM-V-N-A except for the abolished initiation codon of *mpc*. Comparing the expression levels of EscA among these three constructs (Fig 2A, lower panel) revealed that the EscA expression levels from the first two constructs were indistinguishable. However, EscA was detectable but at a relative low level when *mpc* translation initiation was obstructed, given that *escA* is distally separated from *mpc* by two ORFs.

To examine whether the decrease in expression of *escA* is due to an effect of the upstream *escN* gene, a construct with a regular *escN* followed by *escA* was created and the result was pN-A (Fig 2B, upper panel). Similarly, the construct p^*AC*^N-A was created with an A-to-C mutation in the initiation codon of *escN*. Fig 2B (lower panel) shows that *escN* without an effective translation codon did cause a reduction in the expression of *escA* (compare the Western blot from p^*AC*^N-A with that from pN-A). However, the effect was not as strong as that of *escV* on *escN* as seen in Fig 1C. Overall, these results together suggest that the translation initiation of *mpc* is extremely critical to the translation of *escV*, the translation of which is important for the subsequent expression of *escN*. This vicinity translation polar effect of *escN* on the expression of EscA is not as strong as the first two effects (i.e., *mpc* on *escV* and *escV* on *escN*) as outlined above; however, the influence does exist.

### Translation initiation of *mpc* affects the expression of downstream genes far beyond *escP*

**Fig 3:**
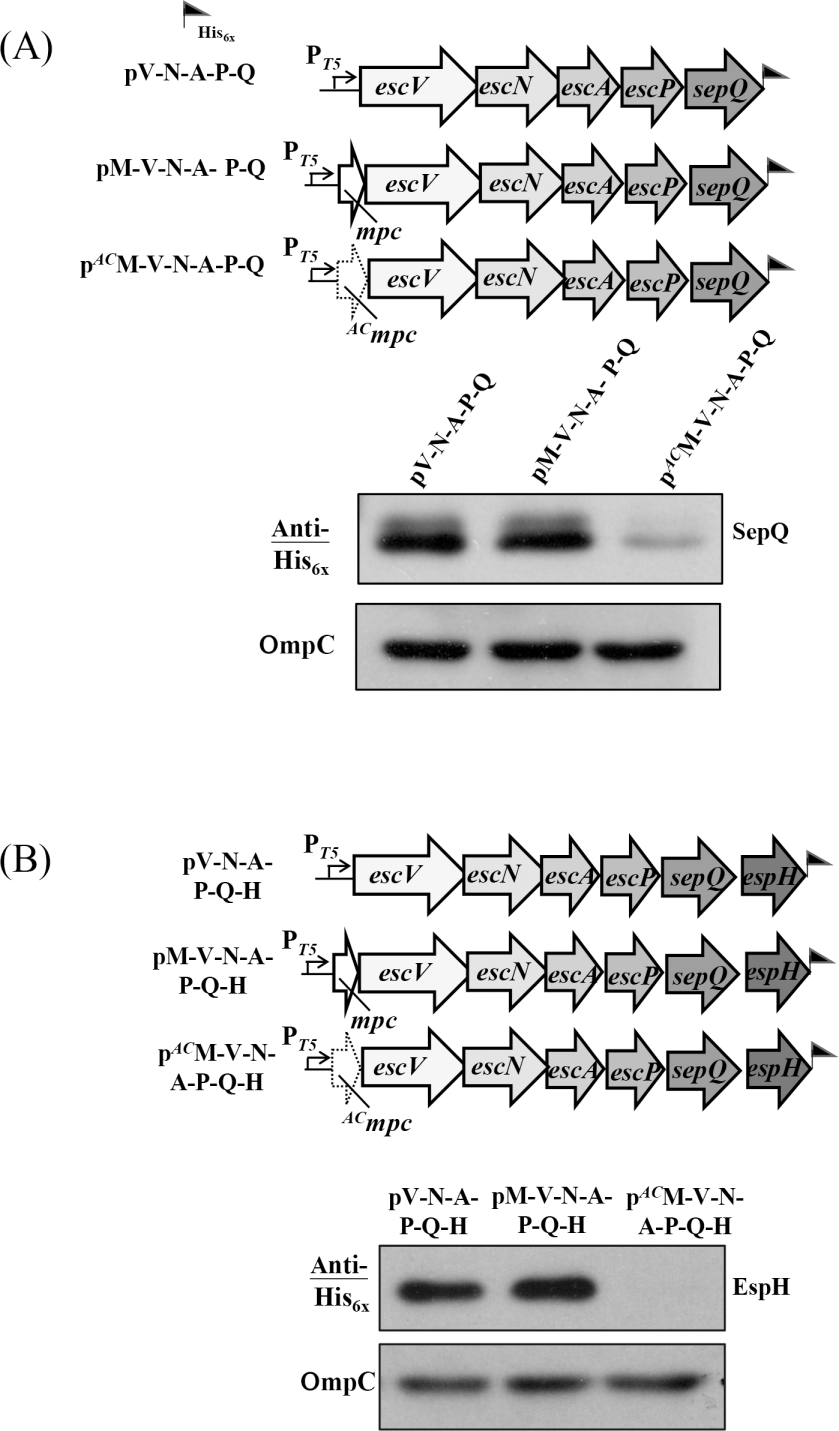
Translation of *mpc* is needed for effective expression of the 6^th^ gene (*sepQ*) and 7^th^ gene (*espH*) of the *lee3* operon. (A) The illustration shows the expression plasmids designed for a comparison of SepQ expression with or without the translation of *mpc*. (B) Comparison of EspH expression with or without the translation of *mpc*. Western blotting analyses are displayed as that described in legend to Fig 1.

To investigate how deep the *mpc* translation initiation effect modifies downstream gene expression, we extended the expression cassette to cover *sepQ* (Fig 3A, upper panel) and *espH* (Fig 3B, upper panel), respectively. Thus, pV-N-A-P-Q contained the segment spanning from *escV* to *sepQ*, while pM-V-N-A-P-Q carried an additional *mpc* in front of *escV* and p^*AC*^M-V-N-A-P-Q carried the same sequence except for a defective initiation codon at *mpc*. Fig 3A (lower panel) shows that the expressed level of SepQ was indistinguishable between pM-V-N-A-P-Q and pV-N-A-P-Q. However, the expressed SepQ decreased severely in p^*AC*^M-V-N-A-P-Q. Therefore, the translation initiation of *mpc* did control the expression of *sepQ* in a manner similar to that seen when *escA* was expressed (Fig 2A). It is worth noting that, when effectively initiated, *mpc* (in pM-V-N-A-P-Q) and *escV* (in pV-N-A-P-Q) made no difference in term of controlling *sepQ* expression. In Fig 3B, pV-N-A-P-Q-H contained the segment ranging from *escV* to *espH* while pM-V-N-A-P-Q-H carried the same segment but had an additional *mpc* in the 5’-end. As shown in the lower panel of Fig 3B, the expression level of EspH was indistinguishable between pV-N-A-P-Q-H and pM-V-N-A-P-Q-H. However, p^*AC*^M-V-N-A-P-Q-H gave a severe reduction in the EspH production when compared with pM-V-N-A-P-Q-H. These results indicate that the effective translation initiation of *mpc* readily is influential to the translation of all downstream *lee3* genes.

### Differences in vicinity effects modulate gene translation

**Fig 4:**
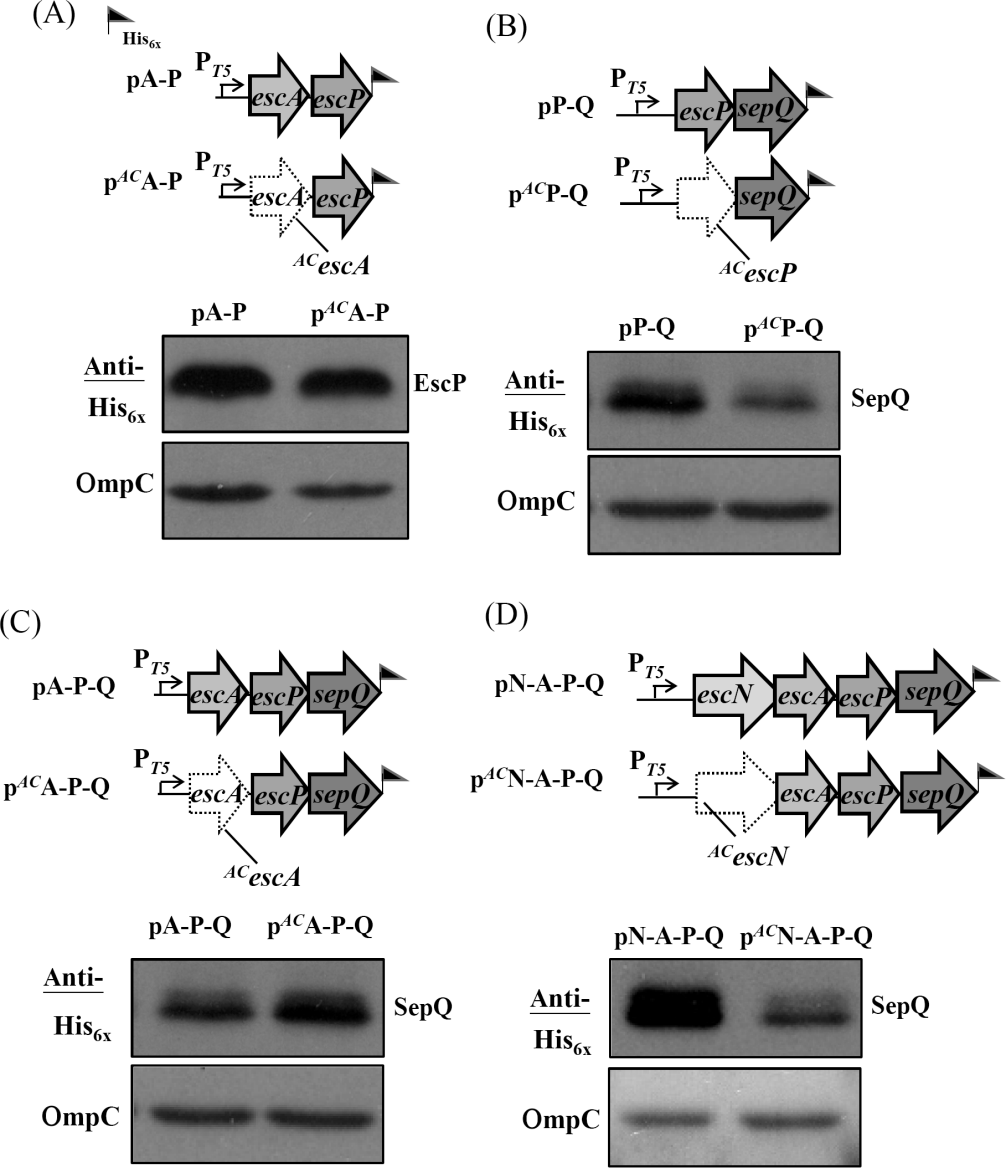
Examining the vicinity effect of translating one gene on the translation of its immediate downstream gene for *escA* and the genes thereafter in *lee3*. (A) Expression of *escP* is reduced only a little by mutating the initiation codon of *escA*, as revealed by comparable levels of EscP. (B) A mild reduction on the amount of SepQ was detected when the translation of *escP* is disrupted by altering the initiation codon of *escP*. (C) Blocking *escA* translation has no effect on the expression of *sepQ*, which is separated from *escA* by *escP* in the middle. (D) Mutation of the initiation codon of *escN* disturbs the expression of *sepQ*. See legend to Fig 1 for explanations of the illustrations, the plasmids used and the Western blotting analysis. Note: the vicinity translation effect of *escN* with respect to *escA* is shown in Fig 2B.

Next we examined whether the enormous vicinity polar effects that affected translation regulation as seen in Fig 1A and 1C involving *mpc* (toward *escV*) and involving *escV* (toward *escN*) also occurred with *escA* (toward *escP*) and with *escP* (toward *sepQ*). To do this, two pairs of constructs were generated. For the first pair, pA-P and p^*AC*^A-P, both contained *escA* and *escP*, where the latter construct carried *escA* with a defective initiation codon (Fig 4A, upper panel). Western blotting was then used to detect the expression of *escP* from the two genetic environments (Fig 4A, lower panel) and the results indicated that they showed very little difference in expression levels after compensating for sample loading using parallel detection of OmpC. A second pair was also compared (Fig 4B, upper panel) in which the examined stretch contained *escP* and *sepQ*. These two plasmids, pP-Q and p^*AC*^P-Q, differed at the first nucleotide of *escP* by one A-to-C mutation. The results of Western blotting showed that the expression level of SepQ from p^*AC*^P-Q was slightly lower than that of SepQ from pP-Q (Fig 4B; lower panel). However, the degree of difference was small if compared to that seen with p^*AC*^M-V (Fig 1A), p^*AC*^V-N (Fig 1C), or p^*AC*^N-A (Fig 2B). Therefore, it would seem that translation of *escA* has only a small effect on the expression of *escP*, and *escP* has a small effect on the expression of *sepQ*. By extrapolation, it is reasonable to speculate that if *escA* is not well translated, it would not affect much the expression of *sepQ*.

To test the above speculation, pA-P-Q and p^*AC*^A-P-Q were constructed (Fig 4C, upper panel). Fig 4C (lower panel) shows the Western blotting results from these two constructs. As expected, SepQ expressed from p^*AC*^A-P-Q was not hampered by the disrupted initiation of *escA* when compared to the result observed with pA-P-Q.

One question that remains is whether extending the above fragment by attaching the upstream *escN*, which does modulate the *escA* expression as shown in Fig 2B, would affect the *sepQ* expression. To address this, pN-A-P-Q and p^*AC*^N-A-P-Q were constructed (Fig 4D, upper panel), and the expression level of SepQ was analyzed accordingly. The Western blotting results in Fig 4D (lower panel) indicate that translation vicinity effect of *escN* toward *escA* (Fig 2B) is readily propelled downstream to cause an obvious reduction in *sepQ* expression. This observation is similar to the polar effect that *mpc* exerting on the expression of *sepQ* in p^*AC*^M-V-N-A-P-Q (Fig 3A).

Finally we examined whether abolishing translation initiation of *sepQ* has any effect on the *espH* expression. We used the same strategy as carried above and did observe that *sepQ* displayed a moderate to strong vicinity translation effect toward *espH* (S1 Fig). Taken together, vicinity effects appear to exist between the neighboring genes of *lee3* in various degrees.

### A *cis* element within *mpc* regulates downstream gene expression

**Fig 5:**
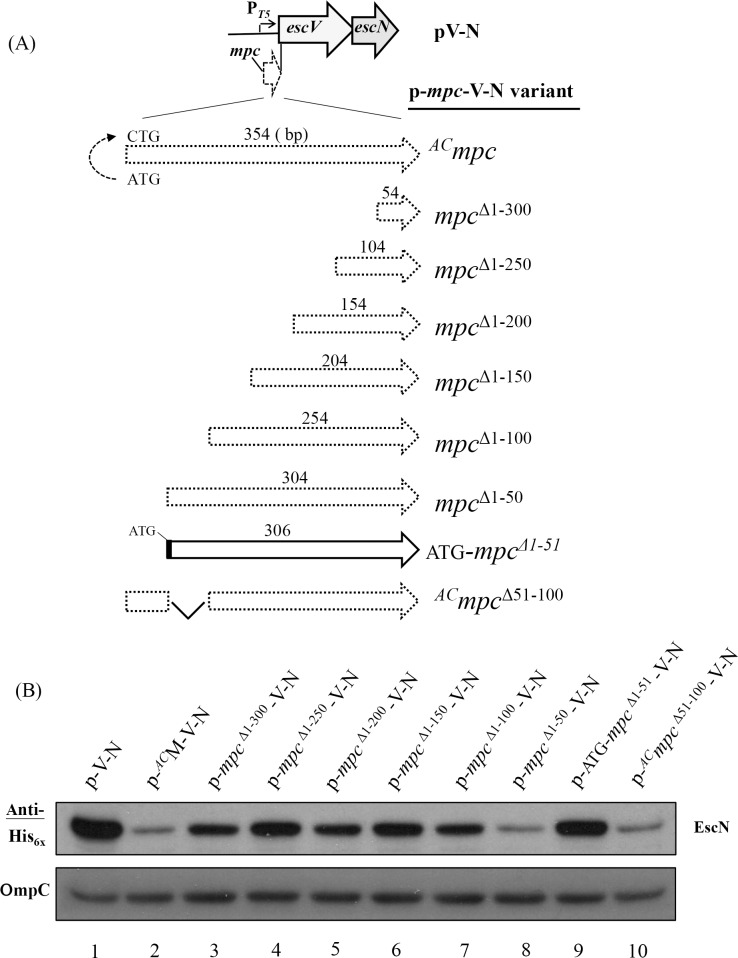
A region spanning the first 100 nucleotides of *mpc* is involved in the expression regulation of the downstream genes within *lee3*. (A) An illustration of the constructs carrying an expression cassette similar to that of pV-N except that differently sized regions from the 3’-end of *mpc* are separately placed upstream to *escV*. (B) Western blotting analysis using anti-His_x6_ is able to detect the amount of tagged EscN expressed by the individual constructs. Note: a dot-framed *mpc* fragment represents the 3’-end remaining *mpc* sequence that has been introduced without an in-frame ATG. The number above each frame represents the size (in base pairs) of the 3’-end fragment of *mpc*. The 5’-end nucleotides of *mpc* truncated are superscripted with “Δ.” A solid-line frame indicates that an ATG has been in-frame engineered in front of the *mpc* fragment containing nucleotides 52–354. Note: *mpc*^Δ51–100^ represents a deletion of nucleotides 51–100 of *mpc* in the construct, and this construct contains no in-frame ATG since the first 50 nucleotides of the frame was derived from ^*AC*^*mpc*. See legend to Fig 1 for additional explanations of the diagram.

To explain why translation of *mpc* is critical for downstream gene expression, one simple hypothesis is that there is a *cis*-element within *mpc* that plays a control role. Its presence may form an as yet undefined structure that hinders immediate downstream gene translation. Thus when ribosomes initiate the translation of *mpc* and read through this element, the transcript then opens this structure so that the downstream gene expressions are facilitated. To explore this possible *cis*-element, we used pV-N and p^*AC*^M-V-N, the results of which were seen above in Fig 1B, for the analysis (Fig 5). Without any *mpc* nucleotide stretch in the sequence, pV-N expressed EscN at a high level as detected by Western blotting in lane 1, Fig 5B. To give a reference of poor expression of EscN, p^*AC*^M-V-N was analyzed in parallel (lane 2). A series of plasmids, named the p-*mpc*-V-N variants in Fig 5A, were then generated in a framework similar to that of pV-N, and into these, the 3’-end of *mpc* was introduced with a gradual increase in size toward the 5’-end. Therefore, p-*mpc*^Δ1-300^-V-N, as an example, represents a construct with nucleotides 301–354 of *mpc* cloned authentically in front of *escV*; in other words, this plasmid was similar to p^*AC*^M-V-N (Fig 1B) but the first 300 nucleotides of *mpc* had been deleted from the authentic segment spanning from *mpc* to *escN*.

Fig 5B shows the Western blotting results when His_x6_-tagged EscN expressed from this series of constructs was detected. Initially, on adding increasing lengths of 3’ *mpc* nucleotides (from the last 54 nucleotides in p-*mpc*^Δ1-300^-V-N up to the last 254 nucleotides in p-*mpc*^Δ1-100^-V-N), it was found that there was no severe reduction in the expression level of EscN compared with the level seen for pV-N (lanes 3–7 *vs*. lane 1, Fig 5B). However, when the 3’-end 304 nucleotides of *mpc* were placed in front of *escV* in the construct of p-*mpc*^Δ1-50^-V-N, *escN* expression was switched off to a very large degree and the level of EscN became as low as that observed for p^*AC*^M-V-N (compare lane 2 with lane 8 in Fig 5B). The *escN* expression bounced back when p-*mpc*^Δ1-100^-V-N had the 5’ region of *mpc* deleted up to 100 nucleotides (lane 7). Therefore, these results together strongly suggest that at least nucleotides 51–100 of *mpc* contain a negative regulation element that prevents downstream genes from expression effectively.

It should be noted that the *mpc* fragment in p-*mpc*^Δ1-50^-V-N starts at nucleotide 51 of *mpc* that does not match a start codon, so initiation is impossible. To make an effective initiation possible so that ribosome is able to read through the putative negative signal, we modified p-*mpc*^Δ1-50^-V-N to give p-ATG-*mpc*^Δ1-51^-V-N in order to provide an in-frame ATG. This construct was then analyzed to determine the level of expression of EscN. As expected and as shown in Fig 5B (lane 9), the expression level of EscN was readily restored to the level as seen in p-*mpc*^Δ1-100^-V-N. These findings suggest that the sequence *per se* at the 5’-end of *mpc*, specifically in nucleotides 51–100, may contain a negative element that is able to repress the ribosomal re-initiation and expression of downstream genes if translation of *mpc* is not initiated.

To examine whether *mpc* nucleotides 51–100 could solely act as a negative element, p^*AC*^*mpc*^Δ51-100^-V-N was constructed in a format similar to the first six 5’-end *mpc* deletion constructs that give no in-frame initiation codon. In this construct, the first nucleotide A of *mpc* was replaced with C while the nucleotides 51–100 were deleted. As show in Fig 5B (lane 10), EscN was expressed from this construct as poor as that from p-*mpc*^Δ1-50^-V-N. Therefore, deleting nucleotides 51–100 of *mpc* did not abolish the 5’-negative suppression signal. Therefore, this suppression element may involve up to the first 100 nucleotides of *mpc*.

Besides the possible *mpc* RNA structure arising from the sequence that may negatively regulate the downstream gene translation, RNA instability owing to a translation blockage could also contribute to the reduction of downstream gene translation. To test this possibility, total RNAs of all transformants of JM109 in Fig 5A were individually harvested and reverse-transcribed into cDNA that was subsequently used for qRT-PCR analysis of the available *escN* RNA. The detected *escN* mRNA levels (S2 Fig) were consistent with the results of proteins identified by Western blotting (Fig 5B). Taken together, these molecular analyses indicates that *mpc* contains a 5’ negative regulatory region affecting the translation of the downstream genes and that a coupled RNA instability also occurs at the downstream region when the translation is not effectively executed at the 5’ distal end.

### Transcription of the mRNA coding for Mpc is at the lowest of all *lee3*-ORFs during T3SS activation

**Fig 6:**
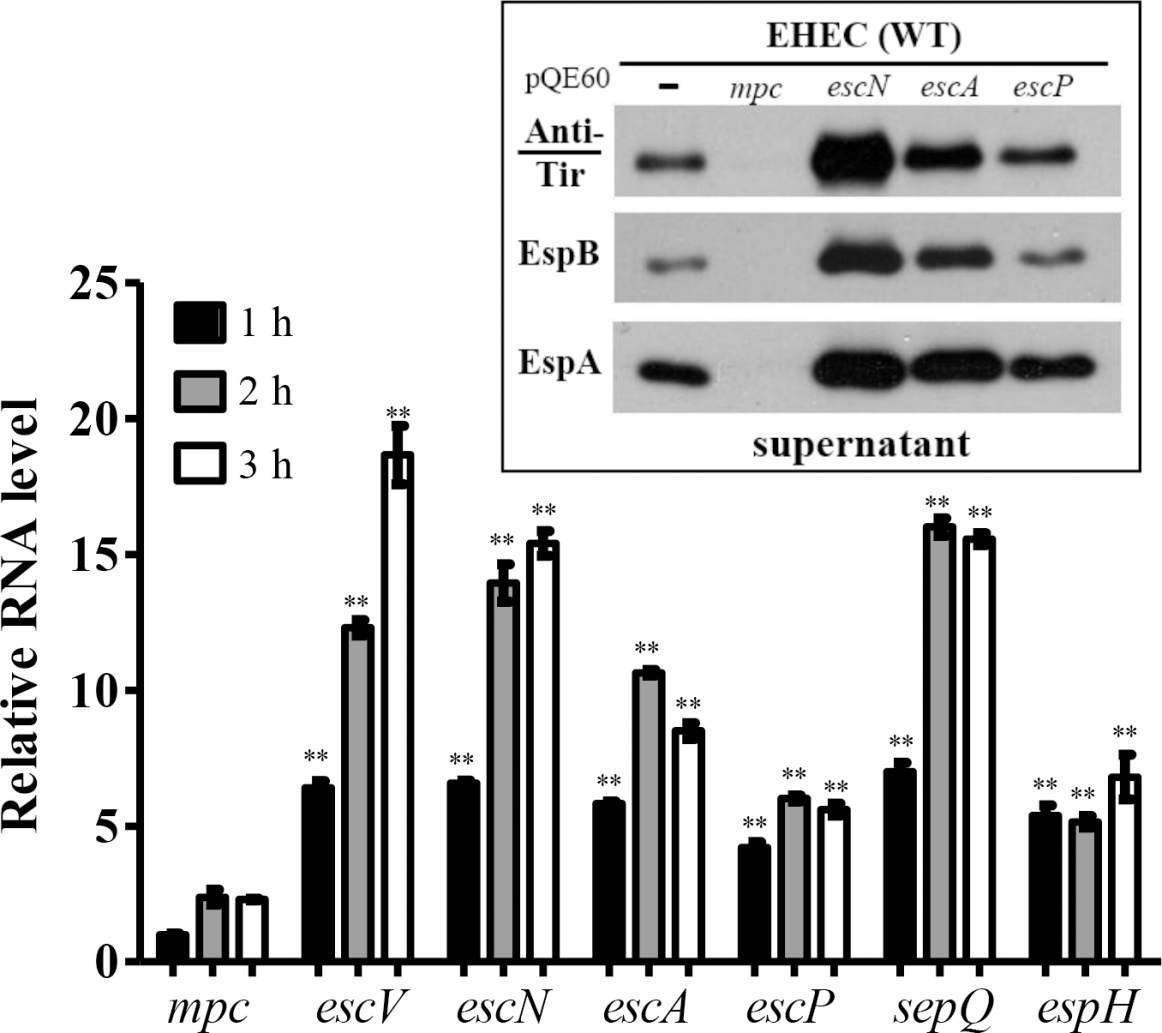
Real-time RT-PCR (qRT-PCR) was used to compare the levels of the RNA transcripts of the ORFs within *lee3* during the process of T3SS activation. EHEC cultivated in LB was switched to M9 in the presence of 5% CO_2_ in order to activate TTSS and then the bacteria were harvested at different time points. Total RNA was isolated, quality assured and then used for real-time RT-PCR. RNA levels were first normalized against the 16S rRNA present in the sample and then their relative amounts (as a fold increase) were calculated against the amount of *mpc* RNA transcript present when detected at hour 1. Experiments were carried out in triplicate, and the qRT-PCR result was further calibrated against the differences of the primer efficiency. Differently painted bars represent the mean values of relative RNA level of genes at different time points while error bars indicate the standard deviations. Student’s *t* test was applied to analyze the significance of the paired data, a comparison referenced accordingly to the measurement of *mpc* (*, *p* < 0.05; **, *p* < 0.01). Note: two different scales are labeled in the Y-axis. **Inset:** protein secretion of TTSS is severely suppressed by over-expression of *mpc* but not by overexpression of *escN*, *escA*, or *escP*. The wild-type strain of EHEC was transformed individually with pQE60-based plasmids to drive target gene expression. After transformation, bacterial culture supernatants were harvested for Western blotting analysis in order to detect secreted Tir, EspB, and EspA by using specific antibodies separately. Over-expressed Mpc, EscN, EscA, and EscP present in the transformants were monitored by analyzing the target proteins present in the appropriate bacterial lysates (data not shown).

Mpc has been suggested to be under stringent regulation [14]. Over-expression of Mpc leads to a strong suppression of T3SS. Furthermore, this suppression is not observed when there is over-expression of *lee3* genes other than *mpc* (Fig 6, inset). We reasoned that there must be one or more additional mechanism that keeps Mpc at a low level. A clue to this was that when doing RT-PCR amplification followed by agarose gel electrophoresis and ethidium bromide staining, we found that, to see comparable band intensities for the *lee3* genes, extra template was needed for the *mpc* amplification. Therefore, at different time points, we harvested samples of EHEC after a medium switch from LB to M9. The total bacterial RNA was then purified, quality confirmed, reverse-transcribed into cDNA, and used for qRT-PCR using individual gene-specific primer pairs. Fig 6 shows the qRT-PCR results. It was found that the detected RNA levels varied between the individual *lee3* ORFs. Specifically, the *mpc* transcript was always the lowest in amount among all seven *lee3* genes at every sampling time. Using the amount detected for *mpc* at the first time point of one hour as a reference, the transcript levels of *escV*, *escN* and *sepQ* were found to be between 6 and 18 fold greater than *mpc*, while the differences for *escA*, *escP* and *espH* were in increases between 4 to 10 fold. These results from sampling at three time points indicated that the detected *mpc* RNA level was the lowest one among those for all *lee3* ORFs. This conclusion was consolidated by that a consistent result was obtained by using alternative primer pairs and freshly isolated samples (S3 Fig). Considering the fact that these genes are in the same operon [9, 14], it is likely that there is a regulatory mechanism that keeps Mpc expression at a low level by restricting the amount of *mpc*-specific mRNA present in the bacterial cell.

## Discussion

In this study, we have uncovered the complexity of regulation taking place within the *lee3* operon and established the role of *mpc* whereby it influences downstream gene expression levels. The regulation within this operon clearly involves multiple layers. First of all, the coding sequence of *mpc* has been documented to enclose binding sites for Ler and H-NS, a fact that suggests the *mpc* DNA sequence *per se* contains a regulatory signal for the binding of positive and negative transcription factors [27, 32]. Secondly, there is another signal spanning the first 100 nucleotides of the *mpc* gene, which appears to contain a negative signal that reduces the expression of the entire operon if translation of *mpc* is not initiated. As a result of these two control mechanisms, the production of gene products that are encoded downstream of *mpc* would be limited under regular LB cultivation. Furthermore, bacteria, when T3SS is activated, do not need much Mpc, which may lead to a shut-down of T3SS (Fig 6) by counteracting Ler when it is over-produced [14]. To achieve this low level of Mpc, a third layer of regulation can be observed that keeps the mRNA needed for *mpc* translation at a low level.

**Fig 7:**
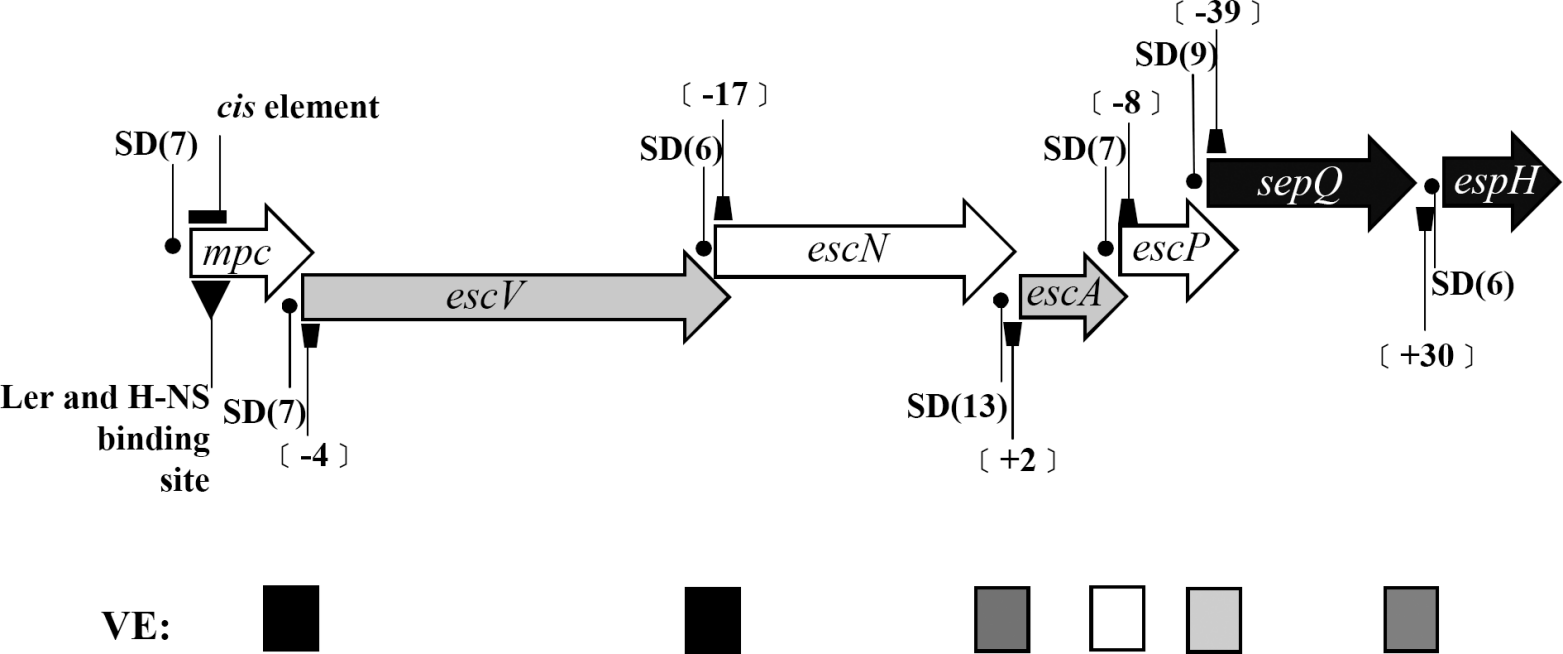
Diagram of the *lee3* operon and the features found therein. The seven genes that form the *lee3* operon are painted differently according to their reading frames. The Shine-Dalgarno sequence (SD) of each gene is marked with a dot and the number in parenthesis following the SD indicates the distance (in nucleotides) between the last nucleotide of the SD sequence and the 1^st^ nucleotide of the next cistron. Keystones represent the distance (in nucleotides) between two neighboring cistrons while negative values indicate an intergenic distance comprising overlapped nucleotides. The inverted and filled triangle below *mpc* marks one of the binding sites of Ler located within *mpc* [27]. The closed box above *mpc* indicates the position of nucleotides 1–100 of *mpc* that have a negative effect on the translation of downstream genes. The filled square marks points where a strong vicinity (polar) effect (VE) is observed with abortive translation of the preceding gene hindering the translation of the following gene, while the open square denotes no such effect being observed; finally, squares in different greyness represents intermediate vicinity effects being observed.

During the elongation step of transcription, current knowledge suggests that the ribosome translates the newly synthesized mRNA in coordination with its rate of synthesis. Deceleration of translation seems to result in a slowdown of RNA polymerase via interactions of NusG and NusE [33, 34]. Such cooperation between RNA polymerase and the ribosome may contribute to a decline in downstream gene transcript production when upstream gene translation is stalled. If we accept this scenario, the transcript of *lee3* with its seven cistrons (Fig 7) would be expected to show comparable qRT-PCR results across the individual cistrons. Our findings indicate that this is apparently not the case and the detected level of the RNA transcript for *mpc* is many fold lower than the level of any of the other cistrons explored. Therefore, there must be a post-transcriptional regulation mechanism that uniquely affects the *lee3* RNA transcript. RNase E has been found to be responsible for cleaving the *lee4* transcript into two derivatives, namely transcripts for *sepL* and *espADB* [35]. Such cleavage allows the two transcripts to be independently translated into their gene products. In *lee3*, translation of *mpc* is detrimental to the translation of downstream genes, a fact suggesting that *lee3* transcript processing and regulation differs greatly from that found of *lee4*. Our simple hypothesis is that the polycistronic RNA transcript of *lee3* may contain specific regulatory structures. One of these structures seems to at least involve the 5’ region of *mpc*. This structure slows down ribosome translation and prevents the over-production of the *lee3* transcripts and, on the other hand, signals for RNase(s) binding/digestion once the ribosome reads through these structures. Such digestion by exonucleases and/or endonucleases at the 5’-region of the mRNA would abort quickly the translational initiation of Mpc and discontinue promptly the generation of other products from the *lee3* transcript.

A few clues to support the above notion are available at the moment and these are as follows. Firstly, initiation of *mpc* translation is vital for the translation of very distally located genes within the same operon, as is the case with SepQ and EspH (Fig 3). Secondly, the first 100 nucleotides of the *mpc* genes are involved in reducing the translation efficiency of the downstream genes, given the first 100 nucleotides of *mpc* are removed from the 5’ end of the *lee3* transcript as would be the case with 5’ exo-nuclease digestion (Fig 5). Thirdly, there are vicinity effects on polar translation of these polycistronic genes whereby poor translation of one gene hinders the effective translation of the following gene; these effects are unevenly segregated within the *lee3* transcript (Fig 7). These vicinity effects seem to associate with the presence of negative *cis*-element on genes, as seen in the case of the first 100 nucleotides of *mpc*. Along this line, our preliminary data found that another *cis*-regulatory element also existed in *escV* (S4 Fig). And additional negative elements of different strengths perhaps would exist in *escN* and *sepQ* as well. Anyway, initiation of *mpc* is necessary for the translation of *escV*, which in turn affects the translation of *escN*. Furthermore, the initiation of translation of *escN* then influences the translation of the next gene, *escA*. Thereafter, however, no strong vicinity effects are seen that affect the translation of the cistrons immediate downstream except for that of *sepQ* toward the *espH* translation (S1 Fig). Anyway, structural interactions of the *lee3* RNA from different vicinities may also play a role to result in the final outcomes of RNAs and proteins detected. On the other hand, there is an alternative model to explain the extremely low level of the *mpc* RNA found among the *lee3* transcripts. It is that unconventional transcription initiation may occur downstream so that the production of the first gene transcript could be skipped. Although this model is less favorable, it could not be totally excluded and needs to be further tested.

Fig 7 summarizes the signals possibly located within the *lee3* operon. The Shine-Dalgarno (SD) sequences are required for ribosome initiative binding, and these SD sequences are identified for individual genes. However, according to SD sequence prediction and based on the distance separating neighboring ORFs, there is no regularity present that could be linked to the identified vicinity effects seen during *lee3* translation. In this regard, all cistrons seem to be equipped with an appropriate SD that is placed at a reasonable distance; furthermore, the overlapping of two reading frames does not necessarily preclude the second gene from being translation by re-initiation [36]. However, it is worth noting that *sepQ* distances from *espH* by 30 nucleotides and within this the SD sequence is found six nucleotides before the initiation codon of *espH*. This genetic organization apparently does not predict whether re-initiation of *espH* translation is possible without the interference of genes earlier in the operon.

Altogether, the evidence for Mpc having multiple functions has been collectively strengthened. It has roles related to counteracting Ler [14], interacting with EscA [17], and, as outlined above, regulating *lee3* operon translation. Furthermore, Mpc has been reported to form complexes with SepL and SepD [37]. SepL and SepD are membrane proteins and play pivotal roles in regulating the secretion hierarchy of translocators and effector proteins. Diminished secretion of translocators, but enhanced secretion of effector proteins, have been observed in *sepL* and *sepD* mutants [38]. This has led to the speculation that production of Mpc may be required for the formation of an integrated Mpc-SepL-SepD complex that ensures correct substrate selectivity. In this context, placing *mpc* at the leading position regarding control of *lee3* may ensure the basal apparatus of T3SS is assembled appropriately at the right time.

## Supporting Information

S1 Fig Vicinity effect seen with *espH* expression when linked authentically downstream to *sepQ*. Disrupting the translation of *sepQ* reduced the expression level of downstream *espH*. The illustrations, the plasmid construction, and the Western blotting analysis were similar to those described in Fig 1.
(TIF)

S2 Fig Quantitative measurement of *escN* mRNA from transformants harboring variants of p-*mpc*-V-N. Plasmids described in Fig 5A were transformed into JM109, and the total RNA was harvested individually from transformants after one hour IPTG induction. RNA was reverse transcribed into cDNA for qRT-PCR analysis of the *escN* mRNA level. Calculation was made after normalizing with the 16s rRNA level and referenced to the value obtained from pV-N for relative amount. (*, *p* < 0.05; **, *p* < 0.01). Note: two different scales are labeled in the Y-axis.
(TIF)

S3 Fig Confirming that the amount of *mpc* RNA is relatively low by qRT-PCR with a second set of primers. Experiments were similarly carried out as that described in Fig 6. However, extract of the EHEC total RNA was re-done after bacteria were activated for T3SS for different periods. Labels of *mpc’*, *escV’* and *sepQ’* denote the results obtained by using different primer pairs but probing the same genes seen in Fig 6.
(TIF)

S4 Fig 5’ region of the *escV* gene negatively regulates downstream gene expression. Generation of the constructs and comparison of the expressed EscA levels were similarly done as in Fig 5 except that the frame of *escN-escA* was authentically extended upstream with increasing lengths of the 3’-end *escV*, of which an intact ORF is consisted of 2028 nucleotides. Note: deleting the 5’end up to 1200 nucleotides of *escV* while keeping the last 828 ones gave no repression of EscA level (lane 7). Deleting less the 5’end nucleotides of *escV*, the suppression effect on the *escA* expression was increasingly seen, particularly with the constructs of *p-escV* ^Δ1–400^—N-A and *p-escV* ^Δ1–200^—N-A (lanes 11 and 12).
(TIF)

S1 Table Primers used and their sequences. (PDF)
