# Supplementary figures and images for: Expression Regulation of Polycistronic lee3 Genes of Enterohaemorrhagic Escherichia coli
Source: PLoS One. 2016 May 16;11(5):e0155578. doi: 10.1371/journal.pone.0155578 (PMC4868261; doi:10.1371/journal.pone.0155578)

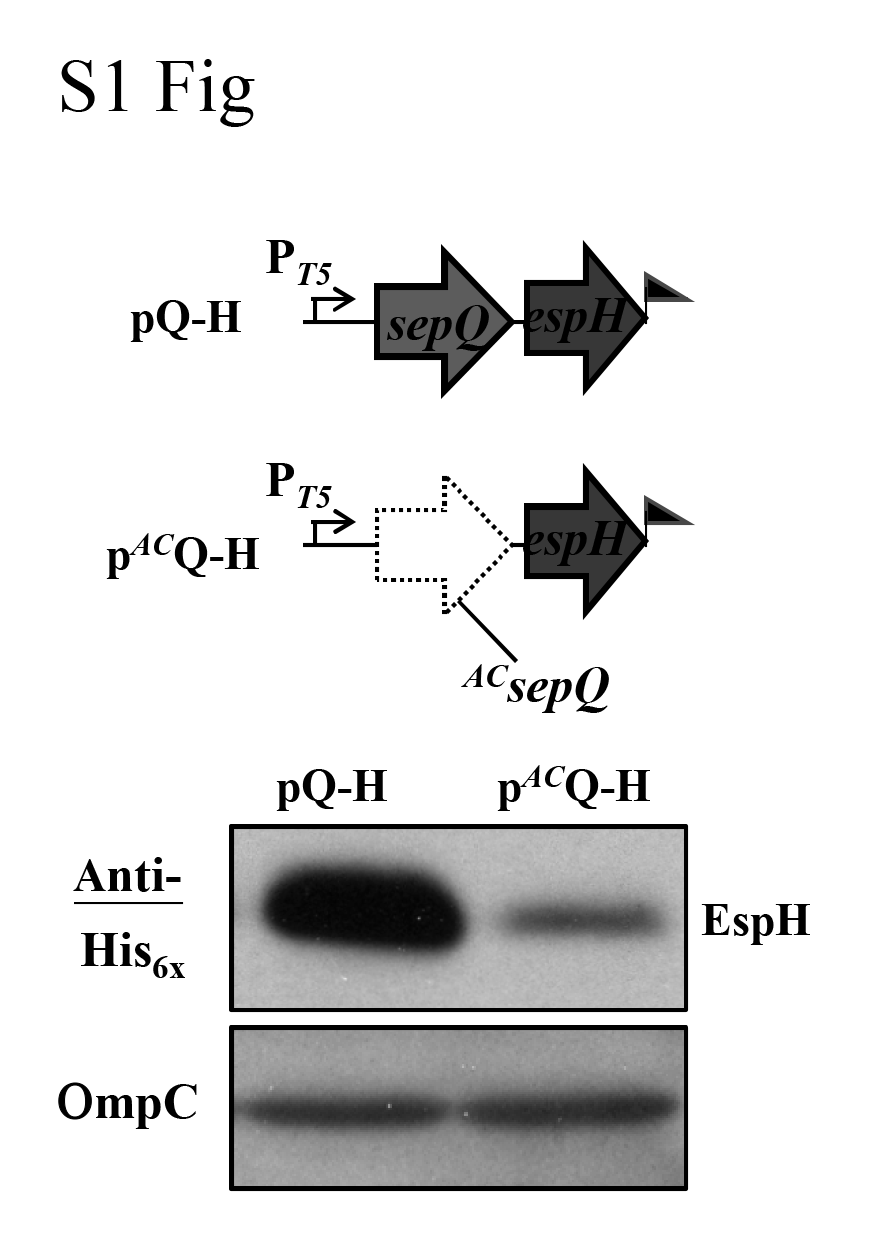

Supplement: S1 Fig — Disrupting the translation of sepQ reduced the expression level of downstream espH. The illustrations, the plasmid construction, and the Western blotting analysis were similar to those described in Fig 1. (TIF) [file pone.0155578.s001.tif]

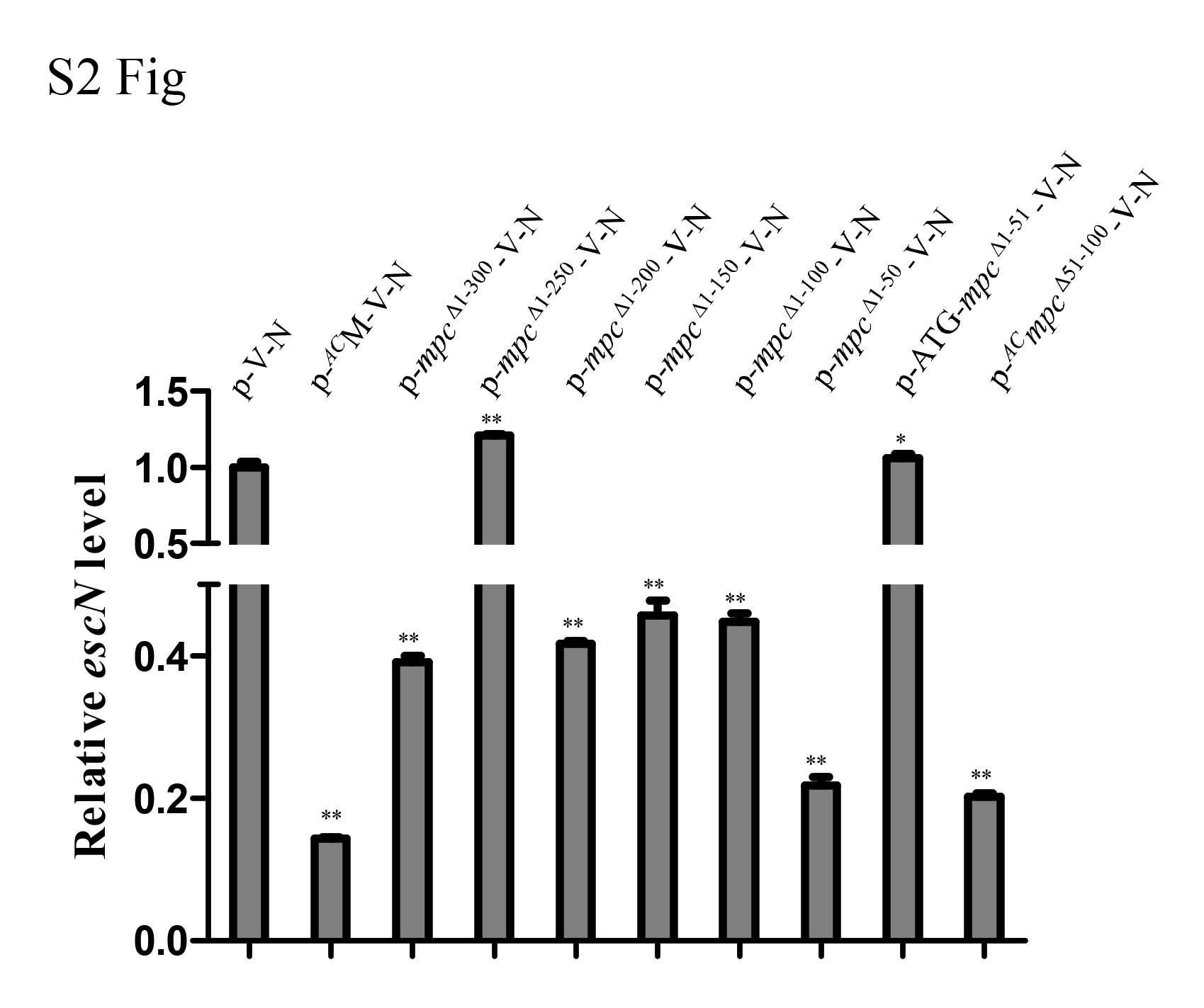

Supplement: S2 Fig — Plasmids described in Fig 5A were transformed into JM109, and the total RNA was harvested individually from transformants after one hour IPTG induction. RNA was reverse transcribed into cDNA for qRT-PCR analysis of the escN mRNA level. Calculation was made after normalizing with the 16s rRNA level and referenced to the value obtained from pV-N for relative amount. (*, p < 0.05; **, p < 0.01). Note: two different scales are labeled in the Y-axis. (TIF) [file pone.0155578.s002.tif]

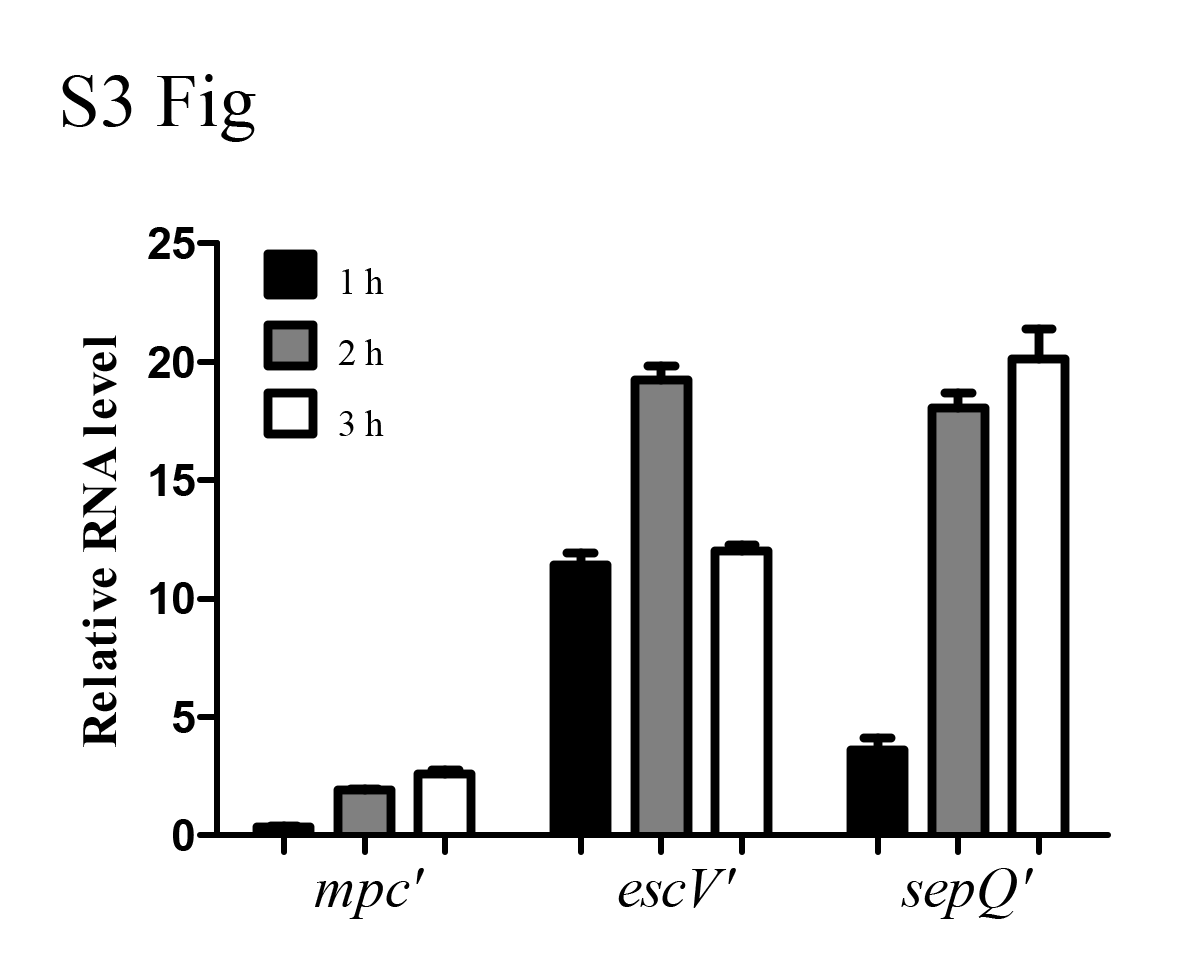

Supplement: S3 Fig — Experiments were similarly carried out as that described in Fig 6. However, extract of the EHEC total RNA was re-done after bacteria were activated for T3SS for different periods. Labels of mpc’, escV’ and sepQ’ denote the results obtained by using different primer pairs but probing the same genes seen in Fig 6. (TIF) [file pone.0155578.s003.tif]

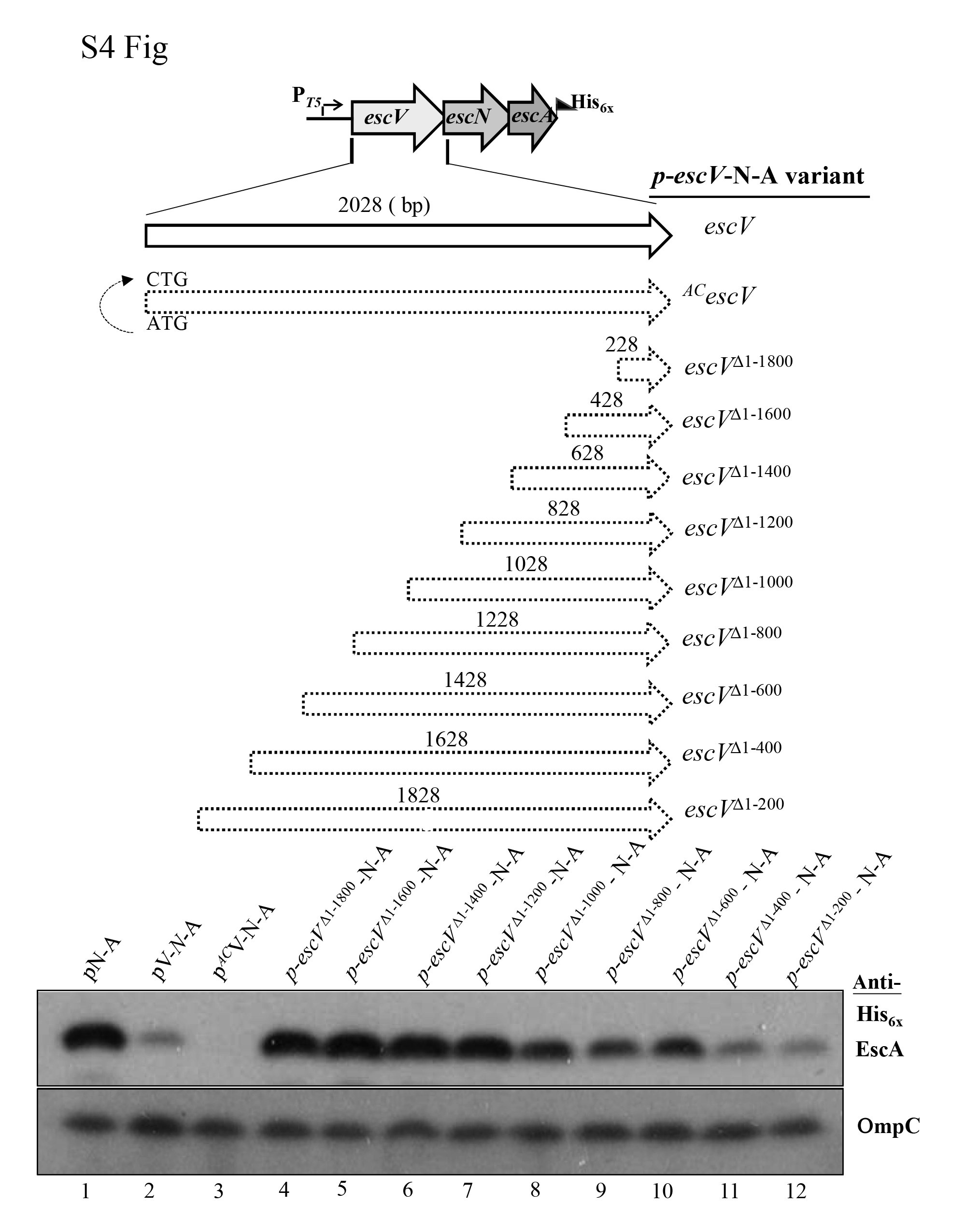

Supplement: S4 Fig — Generation of the constructs and comparison of the expressed EscA levels were similarly done as in Fig 5 except that the frame of escN-escA was authentically extended upstream with increasing lengths of the 3’-end escV, of which an intact ORF is consisted of 2028 nucleotides. Note: deleting the 5’end up to 1200 nucleotides of escV while keeping the last 828 ones gave no repression of EscA level (lane 7). Deleting less the 5’end nucleotides of escV, the suppression effect on the escA expression was increasingly seen, particularly with the constructs of p-escV Δ1–400—N-A and p-escV Δ1–200—N-A (lanes 11 and 12). (TIF) [file pone.0155578.s004.tif]
