# Supplementary material for: Expression Regulation of Polycistronic lee3 Genes of Enterohaemorrhagic Escherichia coli
Source: PLoS One. 2016 May 16;11(5):e0155578. doi: 10.1371/journal.pone.0155578 (PMC4868261; doi:10.1371/journal.pone.0155578)
Supplement: S1 Table — (PDF) [file pone.0155578.s005.pdf]

S1 Table. Primers used and their sequences.

Name	Sequence (5' to 3')	Used for
*Nco*I-*mpc*-F	CCGCCATGGGAATGAATCTTTTAGTTAAAAG	Constructs with *mpc* as the leading cistron; pQE60-*mpc*
*Nco*I-^{AC}*mpc*-F	CCGCCATGGGATGACTGAATCTTTTAGTTAAAAG	Constructs with ^{AC}*mpc* as the leading cistron
*Nco*I-*escV*-F	CCGCCATGGGAATGAATAAACTCTTAAATATATTTAAAAAAG	Constructs with esc*V* as the leading cistron
*Nco*I-^{AC}*escV* -F	CCGCCATGGGATGACTGAATAAACTCTTAAATATA	p^{AC}V-N, p^{AC}V-N-A
*Nco*I-*escN*-F	CCGCCATGGGAATGATTTCAGAGCATGATTCTGTATTG	pN-A, pN-A-P-Q, and pQE60-*escN*
*Nco*I- ^{AC}*escN*-F	CCGCCATGGGATGACTGATTTCAGAGCATGATTCTGTATTGG	p^{AC}N-A , and p^{AC}N-A-P-Q
*Nco*I-*escA*-F	CCGCCATGGGAATGTTGGACAGAATTTTATCTATTCGTAAAAG	pA-P, pA-P-Q, and pQE60-*escA*
*Nco*I- ^{AC}*escA*-F	CCGCCATGGGATGACTGTTGGACAGAATTTTATCTATTCGTAAAAG	p^{AC}A-P and p^{AC}A-P-Q
*Nco*I-*escP*-F	CCGCCATGGGATTGACTAGAGTTTCTCTAAAAAGAAATTTG	pP-Q and pQE60-*escP*
*Nco*I- ^{AC}*escP* -F	CCGCCATGGGATGACTGACTAGAGTTTCTCTAAAAAGAAATTTG	p^{AC}P-Q
*Nco*I-*sepQ*-F	CCGCCATGGGAATGAAGCCATTGAGTTCACAATTG	pQ-H
*Nco*I- ^{AC}*sepQ* -F	CCGCCATGGGATGACTGAAGCCATTGAGTTCACAATTG	p^{AC}Q-H
*Bgl*II-*mpc*-R	CCGAGATCTTGATGTCATCCTGCGAACGC	pQE60-*mpc*
*Bgl*II-*escV*-R	CCGAGATCTTGCTCTGAAATCATTTACCG	pV, pM-V, and p^{AC}M-V
*Bgl*II-*escN*-R	CCGAGATCTGGCAACCACTTTGAATAGG	Constructs with *escN* as the last cistron; pQE60-*escN*
*Bgl*II-*escA*-R	CCG AGATCT GTCAAAGTAATGTTCCTTTATGGC	Constructs with *escA* as the last cistron, pQE60-*escA*
*Bgl*II-*escP*-R	CCGAGATCTATTTTCATATTCAATTGTGAACT	pA-P, p^{AC}A-P, and pQE60-*escP*
*Bgl*II-*sepQ*-R	CCGAGATCTATCACATACTATGCTAACAGTAAAATAAAAATC	Constructs with *sepQ* as the last cistron
*Bgl*II-*espH*-R	CCGAGATCTTAATACGCTATAAGAGGAAGCTCTTGTTG	pQ-H, and p^{AC}Q-H

*Nco*I-*mpc*^{301}-F	CCGCCATGGGATGAACTTTTCAACAGCATGTGCAG	p-*mpc* $^{\Delta 1\text{-}300}$ -V-N
*Nco*I-*mpc*^{251}-F	CCGCCATGGGATGATGTGCTTAAGTTGTTTGTTAACC	p-*mpc* $^{\Delta 1\text{-}250}$ -V-N
*Nco*I-*mpc*^{201}-F	CCGCCATGGGATGATGAGCGTACGCAGGGAGTG	p-*mpc* $^{\Delta 1\text{-}200}$ -V-N
*Nco*I-*mpc*^{151}-F	CCGCCATGGGATGATTAGAGTATAACGAAAACGCATTT	p-*mpc* $^{\Delta 1\text{-}150}$ -V-N
*Nco*I-*mpc*^{101}-F	CCGCCATGGGATGATTTCTTTTTTTATTGAAATAATTGAT	p-*mpc* $^{\Delta 1\text{-}100}$ -V-N
*Nco*I-*mpc*^{51}-F	CCGCCATGGGATGAAAATGATTTTTATTTATTTGATAACC	p-*mpc* $^{\Delta 1\text{-}50}$ -V-N
*Nco*I-ATG- *mpc*^{52}-F	CCGCCATGGGAATGAATGATTTTTATTTATTTGATAACCG	p-ATG-*mpc*$^{\Delta 1\text{-}51}$-V-N
*Nco*I-*escV*^{1801}-F	CCGCCATGGGAGATCGTATCTGAATATCTCACCGGAGC	*p-escV*$^{\Delta 1\text{-}1800}$ -N-A
*Nco*I-*escV*^{1601}-F	CCGCCATGGGATGATGAGAACTATCTTTGAGACGCTTATTTT	*p-escV*$^{\Delta 1\text{-}1600}$ -N-A
*Nco*I-*escV*^{1401}-F	CCGCCATGGGATGATAAGATAACGTTTTTGCTAAAGAAACTTG	*p-escV*$^{\Delta 1\text{-}1400}$ -N-A
*Nco*I-*escV*^{1201}-*F*	CCGCCATGGGATGAGAATTTTTGCTCTATCAAGAGTCAATATAC	*p-escV*$^{\Delta 1\text{-}1200}$ -N-A
*Nco*I-*escV*^{1001}-F	CCGCCATGGGATGAATGCTATGGGAGCTGATTTGTCTAATAG	*p-escV*$^{\Delta 1\text{-}1000}$ -N-A
*Nco*I-*escV*^{801}-F	CCGCCATGGGATGAAGAAGAAAACCTTGCAGGTGATATTG	*p-escV*$^{\Delta 1\text{-}800}$ -N-A
*Nco*I-*escV*^{601}-F	CCGCCATGGGATGAGCTGGTATCATTATTGTTTTAGTTAACC	*p-escV*$^{\Delta 1\text{-}600}$ -N-A
*Nco*I-*escV*^{401}-F	CCGCCATGGGATGAGGGCCGAGCGTGTTGCTGAAG	*p-escV*$^{\Delta 1\text{-}400}$ -N-A
*Nco*I-*escV*^{201}-F	CCGCCATGGGATGATTCTTTCCCCACAATCCTGTTG	*p-escV*$^{\Delta 1\text{-}200}$ -N-A
mpc-RT-F	AGAAACGTTGAAGAGTTTTTAAGATTATTGGG	Detection of *mpc* RNA
mpc-RT-R	CTGCACATGCTGTTGAAAAGTATTCATTAG	Detection of *mpc* RNA
mpc'-RT-F	GGAAATGATTTTTATTTATTTGATAACCGTGTTG	Detection of *mpc* RNA
mpc'-RT-R	AAGCACAATTTATTATCGTAGACAAAAATATGTTC	Detection of *mpc* RNA
escV-RT-F	GGTCATGCGTTGGAATTTCT	Detection of *escV* RNA
escV-RT-R	AAGGTGAGCGAGCTGTTG	Detection of *escV* RNA
escV'-RT-F	ACGTTCGTATTGCCCTGCGTC	Detection of *escV* RNA
escV'-RT-R	AAAGAGAGCACGGGGACTGACG	Detection of *escV* RNA
escN-RT-F	TCGGCGGGACGATTATTAAGGC	Detection of *escN* RNA
escN -RT-R	GCTGCCCAATACCGCAAGTTAGTAG	Detection of *escN* RNA
escA-RT-F	AGCAGAGCGAACCGATTGAGAGAATC	Detection of *escA* RNA
escA-RT-R	TGTGAATCTAGCAATGAACGCTTTTCC	Detection of *escA* RNA

escP-RT-F	TGGCTTCTTTATGGAAGAGATTGTTTTACT	Detection of *escP* RNA
escP-RT-R	AATTGTGAACTCAATGGCTTCATCA	Detection of *escP* RNA
sepQ-RT-F	TCTGGCTGCGGATCTGTTAT	Detection of *sepQ* RNA
sepQ-RT-R	CTCCCGTATGCTATCGTCGT	Detection of *sepQ* RNA
sepQ'-RT-F	TGCCGTTGTTGCCAGTTATCGG	Detection of *sepQ* RNA
sepQ'-RT-F	ACCTTCCGGTAAGGCAGTCTGCA	Detection of *sepQ* RNA
espH-RT-F	CAAGTGAACATGCGTCATGGAACA	Detection of *espH* RNA
espH-RT-R	TCTTTCAGCTAAGATCCCCTCCAGG	Detection of *espH* RNA
bla-RT-F	CATTTCCGTGTCGCCCTTATTC	Detection of *β-lactamase* RNA
bla -RT-R	GGCGTCAACACGGGATAATACC	Detection of *β-lactamase* RNA
mpc-G72stop-F	GCTCCTGAGCGTACGCAGTGATGATTGAACATATTTTTG	$pM^{G72stop}$-V-N
mpc-G72stop-R	CAAAAATATGTTCAATCACTCACTGCGTACGCTCAGGAGC	$pM^{G72stop}$-V-N
RBS-F	AAGCTTTGAGCGGATAACAATTTCAC	$pM^{G72stop}$-V-N
pACYC177-R	CAAGATCCTGGTATCGGTCTGCG	$pM^{G72stop}$-V-N
mpc-trunc51-100-F	TTTCTTTTTTTATTGAAATAATTGATAATAATG	p-*mpc* $^{\Delta 51\text{-}100}$ -V-N
mpc-trunc51-100-R	CCCAATAATCTTAAAAACTCTTCAACG	p-*mpc* $^{\Delta 51\text{-}100}$ -V-N
